# Rodent Modeling of Alzheimer's Disease in Down Syndrome: *In vivo* and *ex vivo* Approaches

**DOI:** 10.3389/fnins.2022.909669

**Published:** 2022-06-07

**Authors:** Clíona Farrell, Paige Mumford, Frances K. Wiseman

**Affiliations:** UK Dementia Research Institute at University College London, London, United Kingdom

**Keywords:** Down syndrome, Alzheimer's disease, Amyloid-beta, tau, mouse model, neuronal loss, neuroinflammation

## Abstract

There are an estimated 6 million people with Down syndrome (DS) worldwide. In developed countries, the vast majority of these individuals will develop Alzheimer's disease neuropathology characterized by the accumulation of amyloid-β (Aβ) plaques and tau neurofibrillary tangles within the brain, which leads to the early onset of dementia (AD-DS) and reduced life-expectancy. The mean age of onset of clinical dementia is ~55 years and by the age of 80, approaching 100% of individuals with DS will have a dementia diagnosis. DS is caused by trisomy of chromosome 21 (Hsa21) thus an additional copy of a gene(s) on the chromosome must cause the development of AD neuropathology and dementia. Indeed, triplication of the gene *APP* which encodes the amyloid precursor protein is sufficient and necessary for early onset AD (EOAD), both in people who have and do not have DS. However, triplication of other genes on Hsa21 leads to profound differences in neurodevelopment resulting in intellectual disability, elevated incidence of epilepsy and perturbations to the immune system. This different biology may impact on how AD neuropathology and dementia develops in people who have DS. Indeed, genes on Hsa21 other than *APP* when in three-copies can modulate AD-pathogenesis in mouse preclinical models. Understanding this biology better is critical to inform drug selection for AD prevention and therapy trials for people who have DS. Here we will review rodent preclinical models of AD-DS and how these can be used for both *in vivo* and *ex vivo* (cultured cells and organotypic slice cultures) studies to understand the mechanisms that contribute to the early development of AD in people who have DS and test the utility of treatments to prevent or delay the development of disease.

## Introduction

### Down Syndrome

Down syndrome (DS) is caused by trisomy of human chromosome 21 (Hsa21). The condition is associated with intellectual disability, craniofacial dysmorphology, increased risk of congenital heart defects, and disorders of the immune system (Antonarakis et al., 2020), however, these features occur with variable levels of penetrance and severity between individuals who have DS (Wiseman et al., 2009). DS is also a significant genetic risk factor for the development of Alzheimer's disease (AD) (Wiseman et al., 2015; Strydom et al., 2018). DS occurs in 1–700 to 1–1,000 live births, and recently there has been an increase in life expectancy for people who have DS due to improved access and advances in medical care in developed countries (Wu and Morris, 2013; Glasson et al., 2016; de Graaf et al., 2017). The increased life expectancy of people with DS means that more people than ever with DS will develop AD within their lifetime; in the UK, dementia is now a leading cause of mortality for adults who have DS (Hithersay et al., 2019).

### Alzheimer's Disease

AD is a neurodegenerative condition and the leading cause of dementia worldwide. Early-onset AD (EOAD), occurring before 65-years, accounts for <5% of AD cases and is caused by mutations in the amyloid precursor protein gene (*APP*), or in the presenilin genes which encode subunits of the γ-secretase enzyme which proteolytically cleaves APP to form amyloid-β (Aβ) (Mendez, 2017). Alternatively, late-onset AD (LOAD) accounts for the majority of AD cases in the general population. Although many risk loci associated with the immune system, lipid metabolism and others have been identified for LOAD, these are not sufficient or necessary to cause AD (Kunkle et al., 2019).

Pathologically, both EOAD and LOAD are associated with the hallmark protein aggregates of AD, including the accumulation of amyloid plaques, composed of the APP cleavage product Aβ, and neurofibrillary tau tangles (NFTs), which precede the development of neuronal loss and brain atrophy. The first phase of AD is considered to be largely asymptomatic and is associated with the deposition of Aβ, first in diffuse plaques, followed by the formation of dense core plaques starting in the temporal cortices (Davidson et al., 2018). Aβ is formed by the sequential cleavage of APP by β-secretase to form a membrane-bound fragment β-C-Terminal fragment (β-CTF) which is then further cleaved by γ-secretase to generate Aβ. This process is thought to occur largely in neuronal endosomes (Das et al., 2016; Chen et al., 2017). In AD, two major isoforms of the Aβ peptide, Aβ_40_ and Aβ_42_, are formed, with Aβ_42_ being more prone to aggregate. Peptide monomers can form higher order oligomers, fibrils, and ultimately extracellular plaques in AD (Sideris et al., 2021; Yang et al., 2022). Following Aβ deposition, NFTs composed of hyperphosphorylated mis-folded tau protein form intracellularly. NFT pathology has been linked to the first clinical symptoms of AD, whereby mild cognitive impairment (MCI) occurs (Parnetti et al., 2012; Betthauser et al., 2020). NFT pathology occurs early in the entorhinal cortex and spreads to other regions of the brain in a defined temporal-spatial pattern which can be classified by Braak tangle staging (Braak and Braak, 1991; Braak et al., 2006). Neuroinflammation is thought to contribute to synapse loss and neuronal degradation in AD, whereby microglia, the resident brain macrophages, change their activation state and function in response to protein aggregates to prune synapses, release pro-inflammatory mediators and no longer undertake key homeostatic functions (Heneka et al., 2015; Hong et al., 2016; Lučiunaite et al., 2020; Leng and Edison, 2021). Together, these processes of protein aggregation and neuroinflammation lead to cell loss and brain atrophy resulting in the clinical manifestations of dementia.

## Alzheimer's Disease in Down Syndrome

### Genetic Mechanism of AD-DS

Phenotypes associated with DS arise due to the aberrant dosage of genes encoded on Hsa21. People with DS have three-copies of *APP*, which is necessary for the development of AD in those individuals (Prasher et al., 1998; Korbel et al., 2009; Wiseman et al., 2015; Doran et al., 2017). Moreover, duplication of the *APP* locus, in the absence of DS, is a rare cause of EOAD (Rovelet-Lecrux et al., 2006, 2007; Sleegers et al., 2006; Hooli et al., 2012; McNaughton et al., 2012). Thus, three-copies of *APP* are sufficient and necessary to drive AD pathogenesis both in people who have and don't have DS. Consistent with this, a third copy of *APP* causes a 1.5-fold or higher level of full-length APP and its cleavage products in the adult brain of people that have DS and in preclinical mouse models (Cheon et al., 2008; Lana-Elola et al., 2021). However, from what age and in which cell types this gene is dosage-sensitive is unclear, as a previous report suggested that APP protein levels are not raised in the young adult brain in a preclinical model of DS (Choi et al., 2009). *APP* is central in the study of AD-DS, however, three-copies of other Hsa21 genes modulate aspects of AD pathology in preclinical animal models (Sheppard et al., 2012; García-Cerro et al., 2017; Naert et al., 2018; Wiseman et al., 2018; Tosh et al., 2021).

### Amyloid-β Pathology

AD pathology in DS broadly progresses in the same temporal pattern as EOAD and LOAD, with Aβ accumulation occurring first, followed by the progression of NFT pathology and neuronal loss (Davidson et al., 2018; Wegiel et al., 2022). However, AD pathology onset occurs decades earlier in people with DS than in the general population (Wiseman et al., 2015; Davidson et al., 2018; Fortea et al., 2021). The first site of Aβ accumulation in AD-DS in the brain is in intracellular neuronal endosomes, which undergo structural changes and enlargement in DS (Gyure et al., 2001; Mori et al., 2002). This accumulation has been observed during fetal development, as early as 28-weeks (Cataldo et al., 2000). Typically however, Aβ accumulation begins in the teens and early-twenties; extracellular amyloid accumulation is seen in the brain parenchyma, beginning as diffuse plaques (made of non-fibrillary deposits), followed by the presence of dense-core plaques over the next decade (Ropper and Williams, 1980; Mann and Esiri, 1989; Lemere et al., 1996; Wegiel et al., 2022). An early site of extracellular Aβ accumulation in AD-DS is in the hippocampus (Leverenz and Raskind, 1998), while in LOAD the first plaques are reported to occur in the cortex (Thal et al., 2002). However, the earliest site of Aβ accumulation detected by positron emission tomography (PET) is in the striatum in AD-DS, consistent with reports for other genetic forms of EOAD (Handen et al., 2012; Annus et al., 2016; Hanseeuw et al., 2018; Zammit et al., 2020). In the brains of individuals who had LOAD, a higher density of Aβ plaques in the cortex is observed compared to AD-DS (Mann et al., 1987, 2018; Egensperger et al., 1999), however, the mean size of plaques in the brain of people who have AD-DS is larger than in LOAD (Armstrong, 2012). Cerebral amyloid angiopathy also occurs with higher frequency in AD-DS than in LOAD (Head et al., 2017; Mann et al., 2018), but microbleeds are thought to occur with the same frequency as in LOAD (Helman et al., 2019). Aβ_42_ deposition occurs earlier than Aβ_40_ in DS, with Aβ_40_ only identified in late-stage dense-core plaques (Lemere et al., 1996; Hirayama et al., 2003). These differences in Aβ pathology between people who had AD-DS and those who had LOAD may be caused by differences in the mechanisms triggering the accumulation of the peptide. In LOAD in the general population, Aβ accumulation is thought to be caused by impaired clearance of Aβ, whilst in AD-DS, increased Aβ production caused by three-copies of *APP* is the cause.

### Neurofibrillary Tau and Other Pathological Changes

Like in EOAD and LOAD, NFT pathology occurs after Aβ deposition in AD-DS in a defined spatial-temporal pattern consistent with Braak tangle staging, starting in the entorhinal cortex, followed by the hippocampus and neocortex (Braak et al., 2006; Davidson et al., 2018; Wegiel et al., 2022). The density of NFTs in the DS brain greatly increases with age (Wisniewski et al., 1985; Davidson et al., 2018; Wegiel et al., 2022). The development of tau pathology is associated with clinical signs of cognitive impairment, as measured by tau PET (Rafii et al., 2017; Rafii, 2019; Lemoine et al., 2020). Other protein aggregates seen in neurodegeneration can also be found in the DS brain during AD-DS. TDP-43 pathology occurs with lower frequency in AD-DS compared to LOAD, but is comparable to that seen in EOAD, which may be caused by differences in the age of onset of disease in the subtypes of AD (Davidson et al., 2011, 2018; Wegiel et al., 2022). α-synuclein-positive Lewy bodies are found in the amygdala of people with DS at a slightly lower level than seen in EOAD (Gibb et al., 1989; Bodhireddy et al., 1994; Prasher et al., 2010; Wegiel et al., 2022), though the cause of this is currently unknown.

### Neuronal Loss

People with DS have a reduced number of neurons in childhood because of developmental differences, and accumulation of Aβ plaques and NFTs are associated with additional neuronal loss in AD-DS. Evidence suggests that neurofibrillary pathology strongly drives atrophy in many brain regions in DS (Wegiel et al., 2022). The pattern of neuronal loss in AD-DS is similar to that in AD, however, people with DS have increased selective loss of neurons from the nucleus basalis of Meynert (Mann et al., 1984; Casanova et al., 1985). Following AD-pathology onset, age and dementia status correlate with decreased neuronal volume, with huge neuronal loss seen in the entorhinal cortex and hippocampus from the 4th and 5th decades of life, followed by later loss of neurons from the amygdala and other regions (Wegiel et al., 2022). Decreased levels of neuronal PET imaging biomarkers, including N-acetylaspartate, are seen when people with DS develop AD, indicating that neuronal loss is coincident with cognitive decline (Lin et al., 2016; Montal et al., 2021).

### Neuroinflammation

Neuroinflammation is thought to make a significant contribution to the transition from pathology to disease in both LOAD and EOAD pathologies, with microgliosis and astrogliosis being identified in close proximity to plaques and tangles (Heneka et al., 2015; De Strooper and Karran, 2016), and gene-association studies identifying variation in a number of genes expressed in immune cells as predictive for disease (Kunkle et al., 2019). Microglial activation, with decreased branching, larger cell somas, and transcriptomic alterations, are seen in people with DS from a young age, with alterations being identified in post-mortem tissue from children as young as 1-year old (Xue and Streit, 2011; Flores-Aguilar et al., 2020; Palmer et al., 2021). As people who have DS age, ramified microglia (thought to have a homeostatic function) become less numerous, and there is an increase in hypertrophic and senescent cells (Xue and Streit, 2011; Martini et al., 2020). The Aβ and NFT load of the cases in these studies is unclear and this could contribute to the differences observed. Other studies have shown that AD-associated neuroinflammation differs in AD-DS compared to LOAD (Wilcock et al., 2015; Startin et al., 2019a; Martini et al., 2020). In the AD-DS posterior cingulate cortex, more ameboid and rod-shaped microglia have been identified compared to AD (Martini et al., 2020). mRNA levels of many pro-inflammatory markers, including IL-1β, TNFα, and IL-6, as well as proteins shown to be raised in activated microglia, CD64 and CD86, are higher in the temporal lobe of people with AD-DS compared to LOAD, despite having a younger age on average (Wilcock et al., 2015). Like in LOAD, plaques in AD-DS are surrounded by activated microglia and are positive for complement proteins, including C1q and C3 (Stoltzner et al., 2000), and activated microglia closely associate with C1q positive neurons (Head et al., 2001). The glial metabolite marker, myo-inositol, measured by proton magnetic resonance spectroscopy, is raised in the brain of people with DS and continues to increase in AD-DS (Lin et al., 2016; Montal et al., 2021). Many inflammatory cytokines, including IL-6, IL-10, TNFα, and IL-1β are raised in the serum of young adults with DS (Weber et al., 2020), and higher levels of IL-1β are seen in the plasma of people with DS compared to LOAD (Startin et al., 2019a). Furthermore, people with DS have higher CSF levels of YKL-40 (chitinase 3-like protein 1), a biomarker of neuroinflammation and astrogliosis, than carriers of EOAD causal mutations, both before and after a dementia diagnosis (Fagan et al., 2021).

People with DS have a perturbed peripheral immune response (Huggard et al., 2020), including increased susceptibility to infection (Hüls et al., 2021; Illouz et al., 2021), autoimmune disorders (Goldacre et al., 2004) and chronic inflammatory conditions (Ferreira et al., 2016). Peripheral or systemic infection can drive neuroinflammation and worsen cognitive decline and pathology in AD (Perry et al., 2007; Cunningham et al., 2009). Priming of microglia can occur in response to peripheral inflammation, with excessive cytokine production, including release of IL-1β and TNFα (Perry and Holmes, 2014). People with DS can experience chronic inflammatory conditions, such as periodontitis, and are susceptible to repeat infection; these systemic effects may be a driver of neuroinflammation and heightened microglial activation in AD-DS compared to LOAD (Kamer et al., 2016).

### Clinical Features and Prevalence of AD-DS

Due to pre-existing intellectual disability in people with DS, accurate diagnosis of AD-dementia has historically been challenging. Recent advances in cognitive assessments, as well as the use of biomarkers, have aided the diagnosis of dementia and mild-cognitive impairment in people with DS. As in EOAD and LOAD, the early signs of dementia in AD-DS include a decline in memory and language skills (Devenny et al., 2002; Krinsky-McHale et al., 2002; Startin et al., 2019b). Early studies in AD-DS suggested that changes to non-cognitive behaviors are more common in the early phase of dementia than in AD; these include apathy, behavioral impulsivity, decreased motivation and increased stubbornness (Nelson et al., 2001; Ball et al., 2008; Oliver et al., 2011); however more recent work has questioned this finding and indicates that memory loss is one of the earliest changes in people who have AD-DS, consistent with findings in the general population (Startin et al., 2019b).

Other clinical features of AD-DS include the onset of epileptic seizures and motor dysfunction (Hithersay et al., 2017). By contrast to LOAD, the majority of individuals with AD-DS develop seizures (Altuna et al., 2021), and onset of seizures in adults with DS is indicative of AD-onset and is associated with more severe AD progression (Lott et al., 2012). Decline in gait is also indicative of AD onset in people with DS (Anderson-Mooney et al., 2016) and symptoms such as dyspraxia, increased incontinence and grasping reflexes can also occur (Visser et al., 1997).

The average age of dementia diagnosis in people with DS is 55 years (Sinai et al., 2018), with death occurring about 4 years later in most individuals (Hithersay et al., 2019). By 60-years, 70% of people with DS will have a dementia diagnosis and by 80-years, virtually all people with DS will have a dementia diagnosis (McCarron et al., 2014, 2017), showing the age-dependant risk.

Unlike in LOAD, sex does not appear to significantly influence the incidence of AD in DS (Coppus et al., 2006; Lai et al., 2020), however, one study has shown an age-associated effect of a higher incidence of AD-DS in men over 60-years (Mhatre et al., 2021). The onset of menopause in women with DS correlates with the onset of AD (Schupf et al., 2003; Coppus et al., 2010), which is younger than in the general population (Cosgrave et al., 1999). As in LOAD, people with DS who carry an E4 allele of *APOE* have earlier changes to amyloid and clinical AD symptoms (Deb et al., 2000; Prasher et al., 2008; Fortea et al., 2020; Bejanin et al., 2021; Lemere et al., 2021).

## Mouse Models of Down Syndrome

Preclinical mouse models have been central to research of the genes and mechanisms that cause the early onset of AD in people who have DS. Several mouse models of DS have been created using a series of genome engineering and other state-of-the-art techniques (Table 1). Others have covered these in-depth (Herault et al., 2017), but those used in AD-DS preclinical research studies will be briefly introduced here.

**Table 1:** Mouse models of Down syndrome and Alzheimer's disease phenotypes they exhibit.

**Model name**	**Genetic change**	**Alzheimer's disease-like Phenotypes**	**References**
Dp(10Lipi-Zbtb21)1Yey Dp(16)1Yey (Yu et al., 2010)	Segmental duplication of Mmu16 *Lipi-Zbtb21* region	**APP biology:** Raised levels of *App*, FL-APP, CTFs, and Aβ from 4-months in hippocampus and cortex. When crossed with the 5XFAD mouse model of AD, Aβ plaque deposition was exacerbated.	Lana-Elola et al., 2021; Zheng et al., 2022
		**Tau biology:** Accumulation of phosphorylated tau in the cortex at 16-months	Lana-Elola et al., 2021
		**Aging dependant neuronal loss:** Loss of basal forebrain cholinergic neurons in the MSN, entorhinal cortex and LC at 16-months	Lana-Elola et al., 2021
		**Neuroinflammation:** Altered microglia morphology, decreased expression of homeostatic markers and increased expression of phagocytic markers. When crossed with the 5XFAD model of AD, microgliosis was exacerbated.	Pinto et al., 2020; Lana-Elola et al., 2021; Zheng et al., 2021
Dp(10Prmt2-Pdxk)1Yey (Dp(10)1Yey) (Yu et al., 2010)	Segmental duplication of Mmu16 *Prmt2-Pdxk* region	**APP biology:** No change reported	Yu et al., 2010
		**Tau biology:** No change reported	
		**Aging dependant neuronal loss:** No change reported	
		**Neuroinflammation:** No change reported	
Dp(17Abcg1-Rrp1b)1Yey (Dp(17)1Yey) (Yu et al., 2010)	Segmental duplication of Mmu16 *Abcg1-Rrp1b* region	**APP biology:** No change reported	Yu et al., 2010
		**Tau biology:** No change reported	
		**Aging dependant neuronal loss:** No change reported	
		**Neuroinflammation:** No change reported	
Dp(16Lipi-Zbtb21)1TybEmcf (Dp1Tyb) (Lana-Elola et al., 2016)	Segmental duplication of Mmu16 *Lipi-Zbtb21* region	**APP biology:** Raised levels of FL-APP, CTFs and Aβ, no Aβ plaques.	Lana-Elola et al., 2021.
		**Tau biology:** No change reported	
		**Aging dependant neuronal loss:** No change reported	
		**Neuroinflammation:** No change reported	
Dp(16Mis18aRunx1)2TybEmcf (Dp2Tyb) (Lana-Elola et al., 2016)	Segmental duplication of Mmu16 *Mis18-Runx1* region	**APP biology:** No change. When crossed with the J20 model of amyloid accumulation, caused a decrease in insoluble Aβ42 levels.	Tosh et al., 2021
		**Tau biology:** No change reported	
		**Aging dependant neuronal loss:** No change reported	
		**Neuroinflammation:** No change reported	
Dp(16Mir802-Zbtb21)3TybEmcf (Dp3Tyb) (Lana-Elola et al., 2016)	Segmental duplication of Mmu16 *Mir802-Zbtb21* region	**APP biology:** No change. When crossed with J20 model of amyloid accumulation, caused an increase in α-CTF/FL-APP ratio, insoluble Aβ42 levels, and oligomeric Aβ levels in the cortex.	Tosh et al., 2021
		**Tau biology:** No change reported	
		**Aging dependant neuronal loss:** No change reported	
		**Neuroinflammation:** No change reported	
Ts(16C-tel)1Cje (Ts1Cje) (Sago et al., 1998)	Translocated Mmu16 segment *Sod1-Mx1* onto Mmu12	**APP biology:** No change reported	Lana-Elola et al., 2016
		**Tau biology:** Increased abundance of phosphorylated tau.	Shukkur et al., 2006; Ishihara et al., 2019
		**Aging dependant neuronal loss:** No change reported	Sago et al., 1998
		**Neuroinflammation:** Increased expression of immune pathway genes.	Guedj et al., 2015
T(171)65Dn (Ts65Dn) (Davisson et al., 1990)	Freely segregating supernumary chromosome containing Mmu16 *Mrpl39-Zfp295* region fused with centromic region from Mmu17	**APP biology:** Raised levels of *App*, FL-APP and Aβ in aging-dependant manner in hippocampus and cortex.	Hunter et al., 2003a; Choi et al., 2009; Netzer et al., 2010; Ahmed et al., 2017; Illouz et al., 2019; Tallino et al., 2022
		**Tau biology:** Increased *Mapt* transcript levels, raised tau protein levels and increased abundance of phosphorylated tau. Alteration to *Mapt* exon 10 splicing.	Liu et al., 2008; Qian et al., 2013; Zhang et al., 2015; Dorard et al., 2016; Ahmed et al., 2017; García-Cerro et al., 2017; Yin et al., 2017; Chen et al., 2021
		**Aging dependant neuronal loss:** Loss of basal forebrain cholinergic neurons in the MSN.	Holtzman et al., 1996; Granholm et al., 2000; Hunter et al., 2003a,b; Seo and Isacson, 2005; Salehi et al., 2006, 2009; Lockrow et al., 2011b; Corrales et al., 2013
		**Neuroinflammation:** Increased density of CD45+ microglia, decreased expression of homeostatic markers and increased levels of poinflammatory cytokines and ROS in aging dependant manner. Astrogliosis in older age.	Hunter et al., 2004a,b; Contestabile et al., 2006; Lockrow et al., 2011a; Ahmed et al., 2017; Rueda et al., 2018; Illouz et al., 2019; Hamlett et al., 2020a
Ts[Rb(12.1716)]2Cje (Ts2Cje) (Villar et al., 2005)	Translocation of Ts65Dn marker chromosome onto Mmu12	**APP biology:** No change reported	Villar et al., 2005
		**Tau biology:** No change reported	
		**Aging dependant neuronal loss:** Loss of basal forebrain cholinergic neurons in the MSN at 9-months	Jiang et al., 2016
		**Neuroinflammation:** No change reported	
Tc(Hsa21)1TybEmcf (Tc1) (O'Doherty et al., 2005)	Freely segregating copy of Hsa21 (mosaic) with 75% of genes expressed	**APP biology:** No change. When crossed with J20 model of amyloid accumulation, caused increased Aβ accumulation.	Wiseman et al., 2018
		**Tau biology:** Evidence of increased tau protein levels and increased abundance of phosphorylated tau.	Sheppard et al., 2012; Ahmed et al., 2013
		**Aging dependant neuronal loss:** No change reported	
		**Neuroinflammation:** No change reported	
Tc(HSA21,CAG-EGFP)1Yakaz/J (TcMAC21) (Kazuki et al., 2020)	Freely segregating mouse artificial chromosome containing Hsa21 with 95% Hsa21 genes expressed	**APP biology:** Raised levels of FL-APP and Aβ from 15-months. Aβ40/Aβ42 ratio is unchanged.	Kazuki et al., 2020
		**Tau biology:** No change reported	
		**Aging dependant neuronal loss:** No change reported	
		**Neuroinflammation:** No change reported	

*FL-APP, Full length amyloid precursor protein; CTFs, C-terminal fragments; Ab, amyloid-beta; AD, Alzheimer's disease; MSN, medial septal nucleus; LC, locus coeruleus; Hsa21, Human chromosome 21; Mmu, mouse chromosome*.

### Segmental Duplication Models

The mouse orthologs of Hsa21 genes are spread across three regions of synteny on mouse chromosomes (Mmu) 10, 16, and 17 (Mouse Genome Informatics, Bult et al., 2019). Chromosomal engineering technology using Cre/*LoxP* recombination has enabled the creation of mouse models which have a duplication of the regions of the mouse chromosomes that are syntenic with Hsa21. These include several models that have been used in AD-DS research, which have different regions of Hsa21 orthologs on Mmu16 in three-copies; for example the Dp(16)1Yey (Yu et al., 2010) and Dp(16Lipi-Zbtb21)1TybEmcf (Dp1Tyb) (Lana-Elola et al., 2016) models with region *Lipi-Zbtb21* in three-copies; Dp(16Mis18aRunx1)2TybEmcf (Dp2Tyb) with region *Mis18-Runx1* in three-copies; and the Dp(16Mir802-Zbtb21)3TybEmcf (Dp3Tyb) with region *Mir802-Zbtb21* in three-copies (Lana-Elola et al., 2016). Mouse models with the Mmu10 or Mmu17 syntenic Hsa21 regions in three-copies include the Dp (10Prmt2-Pdxk)1Yey (Dp10Yey) and Dp(17Abcg1-Rrp1b)1Yey (Dp17Yey) (Yu et al., 2010). The Ts(16C-tel)1Cje (Ts1Cje) mouse model (Sago et al., 1998), which originated from accidental translocation of Mmu16 segments onto Mmu12, has three-copies of the region *Sod1-Mx1* on Mmu16 orthologous to Hsa21.

Using such models with different groups of Hsa21 orthologs in three-copies can be used to determine the combination of genes necessary to cause specific DS phenotypes and the identification of gene candidates. However, many DS phenotypes are multigenic, with many Hsa21 genes required to be in three-copies to result in a phenotype, and there may also be interactions between genes located far away from each other on the chromosome. Such complexities are not modeled when using the smaller segmental duplication models, thus it is important to keep these limitations in mind when interpreting experimental findings.

### Models of Aneuploidy

It is not clear which DS-associated phenotypes are caused by having an extra chromosome (aneuploidy) or by having three-copies of Hsa21 genes. The models discussed above have a duplication on one chromosome of a region of genes; while having three-copies of those genes, they remain euploid. Another approach to modeling DS is to also model the effect of aneuploidy. Models of aneuploidy have also been covered in-depth by others (Tosh et al., 2022), but those used in AD-DS preclinical research will be briefly discussed here. The T(171)65Dn (Ts65Dn) mouse model (Davisson et al., 1990) has a freely segregating, supernumerary chromosome containing the region *Mrpl39-Zfp295* on Mmu16 orthologous to Hsa21 fused with the centromic region of ~10 Mb from Mmu17, resulting in a total of ~90 protein-coding Hsa21 orthologs in three-copies. However, this model also carries three-copies of an additional 35 protein-coding genes on Mmu17 that are not orthologous to Hsa21, thus, gene-dosage effects resulting from these are not relevant to the study of DS. Moreover, phenotypic drift has been recently reported in this model (Shaw et al., 2020), likely caused by its highly complex genetic background. Similarly, the Ts[Rb(12.1716)]2Cje (Ts2Cje) mouse model (Villar et al., 2005), created by Robertsonian translocation of the Ts65Dn's marker chromosome onto Mmu12, contains the same genetic region in three-copies as the Ts65Dn. Both models have been used for a number of AD-DS preclinical research studies.

The Tc(Hsa21)1TybEmcf (Tc1) mouse model (O'Doherty et al., 2005) contains a freely segregating copy of Hsa21. Tc1 mice are mosaic, with ~66% of brain cells carrying the Hsa21, though this percentage varies across tissues. Furthermore, the freely segregating copy of Hsa21 has some deletions and rearrangements, resulting in ~75% of Hsa21 genes being present and functional in the mouse (Gribble et al., 2013). Notably for the study of AD-DS, this model does not contain an additional functional copy of *APP*. Most recently, the Tc(HSA21,CAG-EGFP)1Yakaz/J (TcMAC21) model (Kazuki et al., 2020) was created which carries a mouse artificial chromosome (MAC) with a nearly complete copy of Hsa21; this model is not mosaic because of the use of mouse centromere to carry the human gene-content. While some deletions occurred during generation of the model where ~28% of Hsa21 is missing, only 14 protein-coding genes are within the deleted regions, leaving ~95% of the protein-coding Hsa21 genes intact. Thus, it is currently the most complete aneuploid model of DS and may be a highly useful tool for AD-DS preclinical research.

## Mouse Models of aβ Pathology in Alzheimer's Disease

### AD-Causal Mutations

A range of mouse models exist to study the characteristic amyloid and tau pathology of AD (Götz et al., 2018), here we will briefly describe the amyloid models used in AD-DS preclinical research studies. Mouse Aβ does not readily form extracellular aggregates due to a difference in 3 amino acids between the mouse and human Aβ sequence (Lv et al., 2013). Thus, mouse models that express human Aβ and carry point mutations which cause familial AD in the general population have been used to model Aβ aggregation and accumulation *in vivo*. These include mutations to the *APP* gene, such as the Swedish mutations (Mullan et al., 1992) which are located near the β-secretase cleavage site of Aβ and increase APP processing by β-secretase, elevating Aβ production (Citron et al., 1992). The *APP* Indiana (Murrell et al., 1991) and Iberian (Guerreiro et al., 2010) mutations are located near the γ-secretase cleavage site of Aβ and increase the Aβ_42_ /Aβ_40_ ratio, thus enhancing Aβ aggregation and accelerating plaque formation. The Arctic mutation (Kamino et al., 1992), which changes the biophysical properties of the Aβ and cause the faster formation of protofibrils, has also been used in mouse models. There are also multiple AD-causal mutations to the *PSEN1* gene which encodes presenilin-1, a subcomponent of the γ-secretase complex involved in APP processing into Aβ (Kelleher and Shen, 2017). *PSEN1* mutations increase APP processing by γ-secretase, thus increasing the production of longer, more pathogenic Aβ peptides (Sherrington et al., 1995) and have mostly commonly been used in combination with mutant APP to model aspects of AD in the mouse.

### Transgenic Overexpression *APP* Models

Transgenic overexpression models overexpress human *APP* and/or *PSEN1* carrying familial AD-causal mutations; in these models relatively high expression of the transgene is driven by an artificial promoter, resulting in time-dependent Aβ deposition and coinciding cognitive decline. The J20 model (Mucke et al., 2000) contains the Swedish and Indiana mutations in *APP* and presents robust Aβ plaque deposition from 5 to 7 months of age in the hippocampus and neocortex, with widespread plaques by 8–10 months, and the development of age-dependent deficits in spatial memory and learning (Cheng et al., 2007). It is worth noting that the J20 transgene integrated into the genome in an intron of *Zbtb20*, disrupting its expression (Tosh et al., 2017; Goodwin et al., 2019). ZBTB20 is important to hippocampal development (Mitchelmore et al., 2002), if and how disruption of *Zbtb20* expression contributes to J20 phenotypes requires further study. The 5XFAD model contains three AD-causal mutations in *APP* and two AD-causal mutations in *PSEN1*; Aβ deposition occurs as early as 2-months old, significant Aβ accumulates with age, especially in subiculum and deep cortical layers, and the model also develops age-dependent spatial working memory deficits. Interestingly, the 5XFAD model is one of the few amyloid models to present neuronal loss, especially in brain regions with highest Aβ burden, starting at 6 months (Oakley et al., 2006).

Thus, transgenic overexpression models quickly and robustly present Aβ deposition and coinciding cognitive impairments. Yet, such models have many limitations, including: the highly elevated expression of other APP fragments (secreted APP, β-CTF, α-CTF), each with their own biological functions; the use of artificial promoters to drive transgene expression may result in non-physiological expression of the transgene; and the random insertion of the transgene into a gene locus of the host animal may disrupt endogenous genes (Jankowsky and Zheng, 2017). Furthermore, it has been largely unexplored how the presence of murine APP in these models affects the development of transgene-induced Aβ biology. Indeed, APP/PS1 mice with endogenous mouse *App* knocked-out had elevated levels of aggregated Aβ and higher speed of Aβ deposition than APP/PS1 mice with endogenous mouse *App*, indicating a reduction in the general aggregation propensity of human Aβ when co-existing with endogenous murine Aβ (Steffen et al., 2017).

### Knock-In *App* Models

To overcome some of the limitations of transgenic models, several “knock-in” models have been created which have various familial AD-causing mutations knocked into the murine *App* gene (Saito et al., 2014). In these models, the Aβ sequence is “humanized” (the 3 amino acids in the murine Aβ sequence are changed to match that of the human Aβ sequence), with expression driven by the endogenous murine *App* promoter. As a result, these knock-in models have elevated levels of pathogenic Aβ but physiological levels of FL-APP which is expressed in the correct spatial and temporal context (Saito et al., 2014). Furthermore, knock-in models of wild-type humanized Aβ APP containing no AD-causal point mutations have been created (Serneels et al., 2020; Baglietto-Vargas et al., 2021). Serneels et al. (2020) created both a rat and mouse humanized Aβ APP model and found that humanization increased the production of Aβ_40_ compared to the murine Aβ_40_, coinciding with increased β-CTF and decreased α-CTF levels. Furthermore, a genetically similar humanized *App* mouse model displayed age-dependent decreases in cognitive function, synaptic plasticity, and hippocampal volume (Baglietto-Vargas et al., 2021). Similar humanization of rodent *App* in DS preclinical models will provide the next generation of tools for AD-DS preclinical research.

## Pre-Clinical Modeling of AD-DS: Mechanistic Insights

### APP and Aβ Pathology

Aβ accumulation is a key early event in the development of AD-DS, and initiates the pathological cascade of events that ultimately lead to brain atrophy and AD-dementia (Hardy and Higgins, 1992). Several key research studies in mouse model systems have been undertaken to understand how three-copies of *APP* cause this and how additional copies of other Hsa21 genes modulate this biology.

The Ts65Dn mouse model of DS exhibits raised *App* mRNA levels in the brain from 4-months (Holtzman et al., 1996), but raised levels of full-length APP (FL-APP) and Aβ have been primarily detected in the older Ts65Dn brain in an aging dependant manner, with increases in the hippocampus and cortex seen from 10-months of age (Hunter et al., 2003b; Choi et al., 2009; Illouz et al., 2019; Tallino et al., 2022). Furthermore, Aβ oligomers have been reported to form in the 12-month Ts65Dn hippocampus (Sansevero et al., 2016). Homeostatic processes in the young Ts65Dn brain may modulate APP protein level to compensate for the third copy of *App*, but with age, this process is less effective. Others have seen an increase in APP and Aβ from as young as 4-months (Netzer et al., 2010; Ahmed et al., 2017), but the level was less than the expected 50% increase, and the relative abundance increased with aging, consistent with aging-dependent breakdown of homeostasis contributing to raised protein levels in DS. Differences in experimental methods to quantify APP products may also account for the differing results between these experiments. Accumulation of insoluble Aβ in 15-month Ts65Dn mice has been reported, with raised abundance of thioflavin S+ inclusions compared to euploid controls reported (Illouz et al., 2019). Similarly, another study detected Aβ aggregates in the cerebellum in 12-month Ts65Dn mice (Lomoio et al., 2009). Notably, cerebellar Aβ deposition is not typically seen until late-stage disease in people with AD-DS and only after substantial accumulation is observed in other brain regions (Mann et al., 2018). Moreover, other studies of 20- and 21-month Ts65Dn mice have reported no detectable accumulation of Aβ aggregates (Reeves et al., 1995; Holtzman et al., 1996).

The Dp(16)1Yey mouse model of DS also exhibits raised levels of *App* mRNA, FL-APP, CTFs, and Aβ in the cortex and hippocampus from as young as 4-months, increasing further by 19-months (Lana-Elola et al., 2021). Importantly, the increase in abundance of CTF was greater than the 1.5-fold increase that would be predicted to occur because of an extra copy of *App*; the mechanism for this is not understood and may have considerable implications for the development of AD primary prevention therapy for people who have DS. The TcMAC21 model has increased levels of FL-APP and Aβ, but the Aβ_40_/Aβ_42_ ratio is unchanged compared to euploid mice (Kazuki et al., 2020). No plaque deposition was observed in this model at 24-months, but learning and memory deficits occur from as young as 3-months (Kazuki et al., 2020). Raised levels of APP or Aβ have not been seen in mouse models lacking three-copies of *App*, including the Tc1, Ts1Cje, Dp2Tyb, Dp3Tyb, Dp10Yey, and Dp17Yey models (Salehi et al., 2006; Wiseman et al., 2018; Tosh et al., 2021), consistent with human clinical-genetic data that indicates that three-copies of *APP* are necessary for the development of AD-pathology in people who have DS (Korbel et al., 2009; Doran et al., 2017).

### The Role of *APP* in Cognitive Decline

Many mouse models of DS exhibit neurodevelopmental changes that cause deficits in cognition and changes to behavior (Herault et al., 2017). In addition to these alterations, aging dependent changes to cognition have also been reported in a number of mouse models of DS. Most notably, the Ts65Dn mouse model exhibits progressive cognitive decline (Hunter et al., 2003a; Seo and Isacson, 2005; Illouz et al., 2019), which has been linked to the increased Aβ in the brain. Aβ immunization using DNA or Aβ-fragment approaches successfully lowers soluble and insoluble Aβ toward the level seen in euploid mice, and this is associated with improvements in spatial learning, short-term memory and object recognition (Belichenko et al., 2016; Illouz et al., 2019). However, the response of Ts65Dn mice to the anti-Aβ was lower than euploid controls. Pharmacological reduction of Aβ using γ-secretase inhibition also rescued cognitive deficits in 4-month Ts65Dn mice (Netzer et al., 2010), suggesting that raised Aβ plays an important role in cognitive decline in AD-DS. However, use of another γ-secretase inhibitor in the same model was thought to improve cognition and neuron loss *via* an alternative mechanism by modulating the Sonic-hedgehog pathway, which is also altered in DS (Stagni et al., 2017). Furthermore, γ-secretase inhibition leads to increased production of CTFs (Netzer et al., 2010), which have been implicated in enlargement of endosomes in DS, contributing to perturbed neuronal transport (Filippone and Praticò, 2021). Given recent data indicating particularly highly elevated levels of this fragment in people with DS and preclinical DS mouse models (Lana-Elola et al., 2021), modulation of γ-secretase in people who have DS should be approached with caution.

Aβ is a key target for cognitive decline in AD-DS because it's role is proposed to occur upstream of other changes; other targets have been investigated in the Ts65Dn model, such as estrogen, which may have utility at later stages of disease progression (Hunter et al., 2004b). Notably, the use of bexarotene, a selective retinoid receptor agonist, to reduce Aβ in the hippocampus of the Ts65Dn mouse, worsened cognitive deficits; likely *via* an effect on thyroid function (Vidal et al., 2021), despite leading to Aβ clearance and improvements in cognitive decline in a model of Aβ accumulation (Cramer et al., 2012). Therapeutic targets for AD-DS must take into consideration the affect that trisomy has on AD-pathology and on wider physiology of individuals with DS, and such that effective drugs for AD might not always be suitable for the treatment of AD-DS, because of the specific physiology and co-morbidities of individuals with the condition. Importantly, DS mouse models recapitulate many DS-associated phenotypes and thus can be used to test the interaction of these co-morbidities with lead-candidates for AD primary prevention.

Notable differences occur between DS and Aβ accumulation mouse models. Firstly, levels of β-CTF are particularly raised in DS models; likely because of a combinatorial effect of an extra dose of *App* and other Hsa21 genes. Comparative studies using *App* segmental duplication models are needed to better understand this key biology. In addition, although Aβ levels are raised in DS mouse models, the peptide is not observed to sustainably aggregate within the brain. This is likely to occur because of differences in the processing and aggregation of mouse and human APP and Aβ (Lv et al., 2013); this limitation could be addressed by the humanization of the Aβ sequence in DS mouse models.

### Chromosome 21 Genes, Other Than *APP*, Modulating Aβ Pathology

Although *App* is the primary gene-candidate for altered Aβ levels and associated cognitive decline in AD-DS, work in mouse preclinical systems has demonstrated that other genes on Hsa21, when in three-copies, can modulate Aβ pathology. DYRK1A [dual-specificity tyrosine-(Y)-phosphorylation regulated kinase 1A] is a protein kinase involved in multiple signaling pathways in the brain that are important for normal brain development and physiology. Normalizing the gene-dose of *Dyrk1a* to two-copies in the Ts65Dn mouse model reduced levels of FL-APP and Aβ in the hippocampus and cortex (García-Cerro et al., 2017), showing that this kinase may contribute to the development of AD-pathology in DS.

Further evidence of non-*App* Hsa21 genes modifying Aβ pathology in AD-DS has come from work crossing various DS mouse models with models of amyloid pathology. The Tc1 mouse model, which does not have an additional functional copy of *APP* (O'Doherty et al., 2005), was crossed with the J20 mouse model. This revealed that the extra copy of a gene(s) in the Tc1 model increased Aβ aggregation and Aβ plaque deposition and worsened plaque-associated cognitive deficits (Wiseman et al., 2018). The increased Aβ aggregation corresponded with an increase in the soluble Aβ_42_/Aβ_40_ ratio, independent of a change in γ-secretase activity or rate of extracellular Aβ clearance (Wiseman et al., 2018). To further investigate which gene or genes were modifying amyloid biology, several segmental duplication mouse models of DS were then crossed with the J20 mouse model, each containing smaller subregions of Hsa21 genes in three-copies; the Dp2Tyb, Dp3Tyb, Dp10Yey, and Dp17Yey (Tosh et al., 2021). Three-copies of the Dp3Tyb region, with ~37 genes in three-copies, increased the α-CTF/FL-APP ratio, insoluble Aβ42 levels, and oligomeric Aβ levels in the cortex (Tosh et al., 2021). Notably, duplication of the Dp3Tyb region alone was not sufficient to cause the increase in Aβ deposition as reported in the previous Tc1 study, which has many more genes in three-copies. This could be due to the multigenic nature of DS phenotypes. Alternatively, differences in the function of human and mouse genes may also have contributed to the differences observed. Interestingly, in the same study three-copies of the Dp2Tyb region was found to cause a significant decrease in insoluble Aβ42 levels, although interpretation of this data is confounded by the high mortality of this cross (Tosh et al., 2021).

Recently, the Dp(16)1Yey model crossed with the 5XFAD mouse model exhibited exacerbated Aβ plaque deposition (Zheng et al., 2022), consistent with the findings of Wiseman et al. (2018). The candidate causal gene for increased Aβ burden in 5XFAD mice was identified as *USP25*, which encodes a ubiquitin-specific protease (Zheng et al., 2022). Overexpressing *USP25* in the 5XFAD mouse exacerbated Aβ deposition while conversely, genetic deficiency of *Usp25* in the 5XFAD mouse reduced Aβ deposition. The proposed mechanism by which USP25 modifies Aβ deposition is *via* modification of APP processing; USP25 overexpression in 5XFAD mice increased APP and BACE1 levels while conversely *Usp25* deficiency in 5XFAD mice reduced levels of FL-APP, α-CTFs and β-CTFs (Zheng et al., 2022). This overexpression paradigm is not exactly comparable to DS, when the expression level of genes in three-copies is only ~50% greater expression (Antonarakis et al., 2020). Notably, both the Tc1 and Dp(16)1Yey mouse model contain an additional copy of *USP25* (Gribble et al., 2013) and the gene is significantly upregulated in the adult hippocampus of the Tc1 model (Granno et al., 2019). However, raised levels of full-length APP are not consistently observed in the Tc1 brain or elevated above 1.5-fold in the Dp(16)1Yey model (which also contains 3-copies of *App*), and raised levels of BACE1 are not observed in either model compared to euploid controls (Wiseman et al., 2018; Lana-Elola et al., 2021). Additional preclinical studies to understand the role of three-copies of *USP25* are required to unravel this complexity.

Furthermore, both Wiseman et al. (2018) and Zheng et al. (2022) did not explore how the presence of murine *App* affects amyloid biology development in the 5XFAD and J20 models. As previously discussed, murine APP/Aβ may interfere with human Aβ pathology development (Steffen et al., 2017). Despite these caveats, studies such as these provide evidence that three-copies of non-*App* Hsa21 genes can modify amyloid biology, highlighting the complex nature of AD-DS development. Further research is required to identify the genes and mechanisms responsible for these differences.

### Changes to Tau Biology

In AD-DS, tau pathology occurs after the development of extensive Aβ within the brain, and in the general population Aβ accumulation is proposed to trigger tau misfolding, hyper-phosphorylation, mislocalization and accumulation (Braak et al., 2006; Davidson et al., 2018; Wegiel et al., 2022). Tau is a product of the *Mapt* gene and multiple isoforms of the protein occur; in particular, the relative abundance of the 3-repeat and 4-repeat forms are proposed to cause some genetic forms of frontotemporal dementia, independently of Aβ (Ghetti et al., 2015). Just as in the general population, the cellular and molecular mechanisms that cause tau pathology in AD-DS are not well understood. Research in DS mouse models has provided important insights into how the changes to transcriptional control, cell-signaling, and autophagy may impact on the development of tau pathology in people who have DS, but further research is needed in this important area.

In the Ts65Dn mouse model, increased levels of *Mapt* transcript occur in the brain because of changed transcript stability (Qian et al., 2013; Zhang et al., 2015). Consistent with this, raised tau protein levels have been reported in several regions of the Ts65Dn and Tc1 brain (Ahmed et al., 2013, 2017; García-Cerro et al., 2017), although this has not been observed in all studies (Sheppard et al., 2012; Chaves et al., 2020; Chen et al., 2021). In one report, the level of raised protein increased with age, consistent with observations for other proteins (Ahmed et al., 2017), suggesting that elevated levels of tau may contribute to development of pathology in older adults who have DS. How this relates to a recent report of alterations to the unfolded protein response, RNA translation and proteostasis in the Ts65Dn model (Lanzillotta et al., 2018; Alldred et al., 2021) is unknown and requires further research. A transitory alteration in *Mapt* exon 10 splicing also occurs in Ts65Dn mice at postnatal day 10 which results in a change to the 3-repeat/4-repeat isoform ratio of tau; notably use of a DYRK1A inhibitor corrected this perturbation to tau biology (Yin et al., 2017); whether such changes also occur during neurodegeneration is unknown. In addition, increased relative abundance of phosphorylated tau has been reported to occur in the Ts65Dn, Tc1 and Ts1Cje mouse models in numerous independent studies suggesting that trisomy of Hsa21 robustly enhances phosphorylation of the protein in the mouse (Shukkur et al., 2006; Liu et al., 2008; Sheppard et al., 2012; Dorard et al., 2016; Chen et al., 2021). However, the sites of phosphorylation vary between the studies, with reports of both extensive phosphorylation across the protein (Liu et al., 2008), and more limited phosphorylation (targeting Thr212) in the literature (Sheppard et al., 2012). Differences in the genetics of the mouse models, technical differences in detection and analysis of tau phosphorylation, and housing of the mice may contribute to this. Notably, great care during sample isolation and preparation is required to ensure the preservation of endogenous phosphorylation (Sheppard et al., 2012; Yin et al., 2017). Despite the reported robust increase in phospho-tau in DS mouse models, NFTs have not been detected in these models (Sheppard et al., 2012), consistent with reports for Aβ accumulation in these models (Hashimoto et al., 2019). Increased levels of tau-positive granular deposits have been reported in the Ts65Dn hippocampus (Kern et al., 2011).

A number of genes and mechanisms have been suggested to contribute to these changes in tau biology. Three recent mouse preclinical studies have suggested that raised abundance of APP/Aβ contributes to altered phosphorylation in the Ts65Dn model (Liu et al., 2008; Illouz et al., 2019; Chen et al., 2021). Notably, in the Tc1 mouse model, which does not carry an additional functional copy of *APP*, aberrant tau phosphorylation at position Thr212 has been reported (Sheppard et al., 2012), suggesting that genes on chromosome 21 other than *APP* may also contribute to the altered tau phosphorylation in people who have DS. DYRK1A, the Hsa21-encoded kinase, has been linked in multiple mouse studies to increased phosphorylation of tau particularly at position Thr212; and the abundance of the kinase has been found to correlate with phosphorylation at this site (Chaves et al., 2020). Thus, three-copies of this gene may contribute to the development of tau neuropathology in people with DS. Intranasal treatment of Ts65Dn mice with rapamycin, which targets the mTOR pathway, normalizes raised abundance of phosphorylated tau (Tramutola et al., 2018; Di Domenico et al., 2019), implicating mTOR signaling in altered tau biology in DS. The effect of rapamycin may occur *via* protein phosphatase 2A (PP2A) which has a key role in the dephosphorylation of tau. Indeed, another study has demonstrated that the PP2A inhibitor SET is increased in the Ts65Dn and that this may contribute to raised tau phosphorylation (Dorard et al., 2016). Lastly, a recent study in the Ts1Cje model has linked raised copper levels in the brain with enhanced phosphorylation of tau (Ishihara et al., 2019). The gene or mechanism underlying this intriguing biology has yet to be elucidated.

Differentiating between the effect of trisomy on the formation of tau pathology, from how it changes the response to the pathology, is highly challenging; two recent preclinical studies have started to address this complexity. The first study compared isolates from trisomy 21 and duplication of *APP* iPSC-derived neurons and measured how these altered hippocampal long-term potentiation (LTP) in wild-type mice (Hu et al., 2018). This demonstrated that the key protein responsible for changes to the electrophysiology of the mice differed between trisomy of Hsa21 and *APP* duplication. The second study demonstrated that tau in neuron-derived small extracellular vesicles isolated from adults with AD-DS can induce altered tau biology in the brain of wild-type mice (Ledreux et al., 2021). Further such preclinical studies are required to understand how trisomy of Hsa21 affects both the development of, and response to, tau pathology.

### Development of Neuronal Loss

Neuronal loss occurs during the development of AD pathology in people with DS and this has been modeled in some mouse models of DS. An age-dependent loss of basal forebrain cholinergic neurons (BFCN) in the medial septal nucleus (MSN), as well as loss of norepinephrine neurons in the locus coeruleus, occur in the brain of the Ts65Dn mouse model (Holtzman et al., 1996; Granholm et al., 2000; Hunter et al., 2004a; Seo and Isacson, 2005; Salehi et al., 2006, 2009; Lockrow et al., 2011b; Corrales et al., 2013). Sex differences in the BFCNs of the Ts65Dn model have been identified, with females showing a larger decrease in BFCN number than age-matched males (Kelley et al., 2014). Loss of BFCNs was associated with a compensatory effect in the 12-month Ts65Dn brain, whereby remaining hippocampal neurons had increased ChAT activity compared to younger mice of the same genotype (Seo and Isacson, 2005). The Ts2Cje model exhibits a loss of BFCNs in the MSN at 9-months of age (Jiang et al., 2016) and the Dp(16)1Yey model exhibits loss of BFCNs in the MSN, entorhinal cortex and locus coeruleus in an age-dependent manner, with significant loss seen at 16-months, but not 4-months (Lana-Elola et al., 2021).

Interruptions in nerve growth factor (NGF) transport have been implicated to underlie the loss of BFCNs in DS preclinical models. Intracerebroventricular NGF supplementation to BFCN cell bodies of the Ts65Dn mouse reversed cholinergic degeneration (Cooper et al., 2001), and the loss of BFCNs in the Ts65Dn mouse is associated with a decreased expression of TrkA, a high-affinity NGF receptor (Granholm et al., 2000). NGF is a neurotrophic factor, which acts *via* its receptors, to modulate the maintenance of neuronal populations, including BFCNs. NGF is produced in the hippocampus, where it is endocytosed by MSN BFCNs and then undergoes retrograde transport toward the cell body (Sofroniew et al., 2001). Hippocampal NGF levels are decreased in the aged Ts65Dn brain and NGF retrograde transport is highly disrupted in degenerating BFCNs, correlating with cognitive decline (Cooper et al., 2001; Bimonte-Nelson et al., 2003; Hunter et al., 2003b; Salehi et al., 2006). NGF dysregulation is also seen in people with DS and is thought to be involved in the loss of BFCNs (Iulita et al., 2014).

BFCN degeneration and dysregulation of NGF transport in DS is driven by three-copies of *App*. Ts1Cje mice, which have only two-copies of *App*, have no alteration to BFCN number (Sago et al., 1998) and have only a moderate impairment of NGF transport compared to Ts65Dn mice (Salehi et al., 2006). Normalization of the gene dose of *App* in both the Ts65Dn and Dp(16)1Yey mice rescued the loss of BFCNs, as well as partial rescue of NGF transport in the Ts65Dn model (Salehi et al., 2006; Lana-Elola et al., 2021). Furthermore, drug treatment of Ts65Dn primary cortical neurons with posiphen, to lower the production of FL-APP, rescued deficits in axonal retrograde transport in BFCNs, indicating that it is raised levels of APP or it's cleavage products which are responsible for these deficits (Chen et al., 2021).

Endosomal enlargement, or an increase in the number of neuronal endosomes, has been shown to occur in DS and in mouse models of DS (Botté et al., 2020), and may contribute to BFCN degeneration in an APP-dependant manner. Increased levels of APP cause endosomal enlargement and disrupt NGF trafficking in Ts65Dn BFCNs, which is partially rescued through normalization of the *App* gene-dose or reduction of APP through siRNA targeting (Cataldo et al., 2003; Xu et al., 2016). Increased β-CTF levels correlate with deficits in NGF transport in the Ts65Dn brain (Salehi et al., 2006), and β-CTF is thought to be associated with enlargement of endosomes in DS. Reducing β-CTF levels through reduction of BACE1 activity in the Ts2Cje mouse model prevents endosomal enlargement and the BFCN neurodegeneration in the MSN, hippocampus and cortex (Jiang et al., 2016), showing similar findings as in DS fibroblasts (Jiang et al., 2010; Kim et al., 2016). A recent transcriptomic study of Ts65Dn BFCNs, isolated prior to extensive degeneration, highlighted that alterations to oxidation and phosphorylation, muscarinic M2 cholinergic receptor abundance, and downstream targets of CREB signaling and synaptogenesis signaling, may contribute to degeneration of these cells (Alldred et al., 2021).

Having three-copies of *APP* in DS is necessary for the development of AD-associated cognitive decline, but whether this is due to increased Aβ production, loss of cholinergic neurons, another mechanism, or these working in synergy, is unclear. In addition to the role of extra *APP* in BFCN degeneration and NGF transport disruptions, estrogen and melatonin supplementation have both been shown to improve cognitive deficits and cholinergic neuron loss in the Ts65Dn brain, despite the melatonin treatment not reducing Aβ or APP levels (Granholm et al., 2002; Corrales et al., 2013), showing that multiple pathways may contribute to this aspect of AD-DS pathology. Cholinergic neuronal loss in DS may also be driven by neuroinflammation, as treatment of Ts65Dn mice with the anti-inflammatory compound minocycline or anti-IL17 prevented cholinergic degeneration in the MSN and stopped the microglial activation or raised cytokine abundance, respectively, seen in untreated mice (Hunter et al., 2004a; Rueda et al., 2018).

As well as *APP*, the Hsa21 gene *DYRK1A* has been implicated to play a role in neurodegeneration in AD-DS. Normalizing the gene-dose of *Dyrk1a* in the Ts65Dn mouse model of DS rescued cholinergic neuron degeneration and modulated the density of senescent cells in the cingulate cortex and hippocampus back to euploid levels (García-Cerro et al., 2017), however, how this affected cognitive decline was not studied. Normalizing the gene-dose of *Dyrk1a* in glutamatergic neurons of the Dp(16)1Yey brain improved long-term explicit memory (Brault et al., 2021); this may have been the result of the gene's important role in neurodevelopment and further studies are thus needed to investigate how three-copies of *DYRK1A* may impact cognitive decline associated with AD-DS.

### Development of Neuroinflammation

Microglial and astrocyte activation, and raised levels of proinflammatory cytokines occur in the brain of people with DS compared to euploid matched individuals (Flores-Aguilar et al., 2020; Palmer et al., 2021). These changes progressively worsen with age and the neuroinflammatory profile is different in people with AD-DS than in euploid individuals who have LOAD (Wilcock et al., 2015; Martini et al., 2020). Neuroinflammation occurs in the Dp(16)1Yey model from around postnatal day 22; hippocampal microglia have larger cell bodies, decreased branching, increased expression of CD68, LAMP1, and IBA1 and engulf greater numbers of synapses than their wild-type counterparts (Pinto et al., 2020; Zheng et al., 2021). This microglial phenotype is associated with worsened cognition, loss of neuronal spines and decreased synaptic potential; phenotypes which were rescued upon microglial depletion or anti-inflammatory drug treatment (Pinto et al., 2020). Gene-dose normalization of *App* partially rescued decreased microglial branching in the Dp(16)1Yey model (Lana-Elola et al., 2021). Another Hsa21 gene, *USP25*, has been implicated to contribute to the worsened AD-DS neuroinflammation in the Dp(16)1Yey mouse, whereby overexpression of this gene in the 5XFAD mouse model of AD caused increase microglial activation, loss of neuronal spines and worsened cognition (Zheng et al., 2021); as previously mentioned, this may occur indirectly *via* an effect on APP processing (Zheng et al., 2022).

The Ts65Dn mouse model exhibits neuroinflammation in an aging-dependant manner. There is an increased density of CD45+ microglia in the hippocampus and basal forebrain of the 10- and 18-month Ts65Dn model (Hunter et al., 2004a; Lockrow et al., 2011a; Hamlett et al., 2020a). IBA1 upregulation in the 12-month hippocampus occurs (Rueda et al., 2018), as well as decreased expression of the homeostatic microglial marker, P2RY12, in the 15-month Ts65Dn (Illouz et al., 2019). Increases in proinflammatory cytokine levels, including IL-1β, IFNγ, IL-17, and GM-CSF, occur in the 12-month hippocampus (Ahmed et al., 2017; Rueda et al., 2018), and serum-levels of proinflammatory markers IL-1, TNFα, and IL-6 are also increased in the 10-month Ts65Dn (Hamlett et al., 2020a). Ts65Dn mice have increased levels of reactive oxygen species in the cortex, which is further associated with BFCN degeneration and memory deficits (Lockrow et al., 2009). Upregulation in genes functionally associated with oxidative stress and immune pathways are elevated in the hippocampus of the Ts1Cje mouse (Guedj et al., 2015). Intriguingly, an exaggerated microglia response occurs in the Ts65Dn brain in which BFCN degeneration has been induced compared with euploid controls, consistent with the hypothesis that trisomy of Hsa21 perturbs neuroinflammation (Hamlett et al., 2020b). The use of anti-inflammatory or anti-oxidant treatments in the Ts65Dn model, including treatment with minocycline, vitamin E, resolvin E, granulocyte-macrophage colony-stimulating factor or anti-IL-17, rescues some of these neuroinflammatory phenotypes, neurodegeneration and cognition (Hunter et al., 2004a; Lockrow et al., 2009; Rueda et al., 2018; Hamlett et al., 2020a; Ahmed et al., 2022), showing the important role that neuroinflammation may have in AD-DS phenotypes.

Astrogliosis occurs in DS and there is thought to be a greater number of astrocytes in the DS brain due to a gliogenic shift that takes place during neurodevelopment (Lu et al., 2011; Ponroy Bally and Murai, 2021). Astrocytes become reactive in neurodegenerative disease, including in AD, and this has been shown to be potentially neurotoxic (Liddelow et al., 2017). Astrocytes in the 15–19-month Ts65Dn hippocampus have increased expression of GFAP, S100β and C3, indicating they have taken on a reactive phenotype (Holtzman et al., 1996; Contestabile et al., 2006; Illouz et al., 2019). Immunization with anti-Aβ reduced the expression of C3 in this model, indicating that this astrogliosis is likely in response to raised Aβ (Illouz et al., 2019). Interestingly, although the key astrocytic gene *S100*β is in three-copies in people with DS, the Ts65Dn model has only 2-copies of this gene (Reeves et al., 1995), and thus the increased expression is due to astrocyte reactivity, rather than an extra gene dose.

Neuroinflammation appears to differ between DS and Aβ accumulation mouse models. Neuroinflammatory responses have been reported in some mouse models of amyloid accumulation at the later stages of progression when substantial misfolded protein has accumulated within the brain, particularly in models that produce human Aβ carrying the Arctic mutation (Saito et al., 2014; Castillo et al., 2017). In contrast, in the Ts65Dn and Dp(16)1Yey model, mouse Aβ, although raised, does not accumulate or deposit within the brain; likely because of differences in the processing of mouse and human APP and different aggregation tendencies of Aβ in the two species (Lv et al., 2013) but an early and significant neuroinflammatory phenotype has been reported. Side-by-side studies are required to verify the observation that despite lower abundance of aggregated Aβ, DS mouse models exhibit elevated neuroinflammation; suggesting that genes on Hsa21 other than *APP* are likely to contribute to the perturbed neuroinflammatory phenotype seen in people who have AD-DS.

Like people with DS, mouse models of DS exhibit alterations in the peripheral immune response, and neuroinflammation may be mediated by peripheral or systemic inflammation. The Ts65Dn model exhibits an altered immune response, with decreased numbers of peripheral immune cells and increased production of ROS (Lorenzo et al., 2013), and the Dp(16)1Yey mouse exhibits peripheral interferon hypersensitivity and increased proinflammatory cytokine production (Tuttle et al., 2020). Ts65Dn and Dp1Tyb mice also develop otitis media (Han et al., 2009; Lana-Elola et al., 2021), or inner ear inflammation, as do many people with DS (Maris et al., 2014); this inflammation may contribute to neuroinflammatory priming in the brain of this model.

In current mouse models of DS, excluding the Tc1 due to its mosaicism, all cells and tissue are trisomic, making it difficult to understand the role of peripheral vs. central effects. The size of the genetic changes in some of these models means that it is not possible to use standard techniques to address this issue, and novel approaches such as iPSC chimera and blastocyst complementation are required to create novel models to address these questions (Chang et al., 2018; Real et al., 2018). Furthermore, it is difficult to study how peripheral infection may contribute to neuroinflammation in mouse models, as modern animal housing conditions often ensure that animals are free of a defined range of pathogens and commensal organisms. Artificial systemic inflammation can be employed to address this issue but may cause adverse welfare outcomes and mortality in DS model systems (Tuttle et al., 2020). This is a potential future route of investigation for understanding the complexities of neuroinflammation in DS.

## *In vitro* and *ex vivo* Rodent Modeling of Down Syndrome

Although mouse models of DS readily model many facets of the syndrome, some phenotypes are subtle and hard to interrogate *in vivo*. Rodent derived primary cell and tissue cultures have been established which allow single cell-types or whole pieces of brain tissue to be studied and manipulated, and these can give great insights into molecular and cellular processes in DS and AD-DS, allowing mechanisms to be explored independent of the confound of peripheral systematic inflammation, for example.

### *In vitro* Modeling

Primary brain cell cultures isolated from animal models are useful for determining the cellular and molecular mechanisms of both health and disease. Primary neuronal cultures are a commonly used tool to investigate neurological disease processes and have been extensively used in AD research. Primary cortical and hippocampal neurons from the Ts65Dn model exhibit aspects of AD and DS neurobiology, including raised levels of APP and CTF production (Chen et al., 2021), enlarged endosomes and disrupted axonal retrograde transport (Xu et al., 2016; Chen et al., 2021). Cortical neurons of the Ts2Cje mouse also exhibit raised APP levels and lysosomal dysfunction (Jiang et al., 2019). Alterations in GABA signaling have been observed in Ts65Dn primary neurons, as have been seen *in vivo* (Best et al., 2007; Lysenko et al., 2018). Neuroinflammation contributes hugely to AD and DS, however, unlike in AD, few studies to date have used primary-cultured astrocytes or microglia from DS mouse models to investigate how these cell types may differ due to trisomy. Although single-cell cultures are able to replicate *in vivo* phenotypes of those seen in people with AD-DS, recent studies have shown that single-cultured brain cells can have altered transcriptomic and proteomic profiles compared to neurons, astrocytes and microglia co-cultures, which express a more *in vivo*-like profile (Delbridge et al., 2020; Baxter et al., 2021). Therefore, although primary cultures can allow the simplified study of cellular mechanisms of brain cells, careful interpretation of results is necessary, as single-cultured cells do not represent cellular interactions normally seen in the brain. Despite their limitations, there are many benefits to primary cultures, and they can particularly be used in AD-DS research to understand how trisomy disrupts cell-autonomous processes. Primary cultures offer benefits according to the 3R's principals of replacement, reduction and refinement as well. Use of primary cultures can reduce the number of rodents required for an experiment compared to *in vivo* studies, can reduce variability due to animal-to-animal variation, and allow for treatments with noxious stimuli, which would be invasive *in vivo*, and which may cause adverse welfare outcomes.

### *Ex vivo* Modeling

Although single-cell cultures can give insight into cellular mechanisms, in the brain, neurons, astrocytes, microglia, oligodendrocytes and other cell types are present in the parenchyma. To try to model these cell-cell interactions, and work with cells in a more complex environment, organotypic brain slice cultures (OBSC) have begun to be used in research for AD, other neurodegenerative diseases, and stroke (Li et al., 2016). OBSCs are an “*ex vivo”* model system involving the maintenance of thinly sliced brain sections in culture (Humpel, 2015). OBSCs maintain the cytoarchitecture seen *in vivo* and contain all major cell types present in the brain, with cell populations representative of that *in vivo* (Staal et al., 2011). OBSCs are representative of *in vivo* cell-cell interactions, are anatomically like the brain region of interest, and have functioning synapses (Croft et al., 2019). OBSCs also offer 3Rs benefits. Multiple slices prepared from the same animal can be used for different treatments, reducing the number of animals required for experimentation and reducing inter-animal variability (Durrant, 2020). Furthermore, as with primary cultures, slices can be easily treated with noxious stimuli through addition into culture media, rendering challenges not possible *in vivo* in DS models because of welfare and mortality to be investigated.

OBSCs have been used to study neurodegenerative diseases, including AD. The pathogenesis of Aβ and tau proteins have been studied in slice cultures from transgenic mouse models, elucidating early mechanisms in protein accumulation (Harwell and Coleman, 2016; Croft et al., 2017; Miller et al., 2021). OBSCs have also been used to investigate neuroinflammation and seem to be a better model than single-cultured microglia, as OBSC-microglia have greater transcriptional and phenotypic similarity to acutely isolated and *in vivo* microglia (Delbridge et al., 2020). Stimulation of OBSCs with LPS resulted in the loss of synaptophysin, showing active microglial phagocytosis in slices (Sheppard et al., 2019), as well as release of proinflammatory cytokines (Hellstrom et al., 2005). Furthermore, NLRP3 inflammasome activity can be induced in OBSCs (Hoyle et al., 2020), showing that microglia can respond to stimuli similarly as seen *in vivo* and that OBSCs offer a useful model to study neuroinflammation in mouse models of neurological disease. OBSCs have been prepared from the brain of the Ts65Dn mouse model to examine synaptic connectivity of hippocampal CA3 neurons (Hanson et al., 2007). Alterations to excitatory and inhibitory synaptic function were identified, similar to what has been seen *in vivo* in this model (Siarey et al., 1997), and in people with DS (Contestabile et al., 2017). This study shows that OBSCs from DS models can recapitulate *in vivo* phenotypes and offer a model system for the future study of AD-DS mechanisms.

## Conclusions

AD-DS has a unique and important place in AD research. The genetic cause of the increased risk of disease is fully established, a large number of individuals with the condition occur in the population, and their lifetime risk of AD is extremely high (Fortea et al., 2021). AD primary prevention trials in people who have DS are feasible, can be targeted at the earliest phase of disease, and thus, have high chances of success (Strydom et al., 2018). However, given the different biology of individuals who have DS, drug selection for these studies needs careful consideration to ensure both safety, and best chance of trial success. Current rodent models of DS have excellent construct validity and exhibit a range of AD relevant phenotypes. Notably, elegant work in preclinical models has already indicated that careful thought is required regarding the use of γ-secretase modulators in people who have DS (Lana-Elola et al., 2021), that bexarotene may clear Aβ effectively but also impairs cognitive performance in a DS preclinical model (Vidal et al., 2021), and that different vaccination protocols for anti-Aβ studies (Illouz et al., 2019) may be required in the context of trisomy of Hsa21.

Moreover, in recent years significant progress has been made to understand the differences and similarities that occur between AD-DS and other forms of AD and have highlighted that disease onset and progression is modulated by many processes. An impressive preclinical toolkit is available to unravel the complex multigenic-multi-cellular interactions that underpin this biology. Novel approaches including humanization of critical genes in DS rodent models, such as *App*, and iPSC-mouse chimeras are important to further our understanding of the development of neuropathology, the transition from pathology to disease and the efficacy and safety of potential treatments, in the context of an additional copy of chromosome 21. Future research using the next-generation of AD-DS rodent models will provide significant insight into which strategies have the best chance of preventing or delaying the development of AD in people who have DS.

## Author Contributions

All authors drafted sections of the manuscript, reviewed, edited the final draft, and read and approved the final manuscript.

## Funding

FW and PM were supported by the UK Dementia Research Institute which receives its funding from DRI Ltd., funded by the UK Medical Research Council, Alzheimer's Society and Alzheimer's Research UK (UKDRI-1014), and FW also holds an Alzheimer's Research UK Senior Research Fellowship (ARUK-SRF2018A-001). CF was funded by an Alzheimer's Society PhD Studentship Awarded (AS-PhD-19a-007) to FW. FW also received funding that contributed to this paper from the MRC via CoEN award MR/S005145/1.

## Conflict of Interest

The authors declare that the research was conducted in the absence of any commercial or financial relationships that could be construed as a potential conflict of interest.

## Publisher's Note

All claims expressed in this article are solely those of the authors and do not necessarily represent those of their affiliated organizations, or those of the publisher, the editors and the reviewers. Any product that may be evaluated in this article, or claim that may be made by its manufacturer, is not guaranteed or endorsed by the publisher.
